# Hemostatic Activity of Canine Never-Frozen Liquid Plasma Collected for Transfusion

**DOI:** 10.3389/fvets.2022.731617

**Published:** 2022-02-15

**Authors:** Daniela Proverbio, Roberta Perego, Luciana Baggiani, Eva Spada

**Affiliations:** Veterinary Transfusion Research Laboratory (REVLab), Department of Veterinary Medicine (DIMEVET), University of Milan, Lodi, Italy

**Keywords:** canine, coagulation, liquid plasma, refrigerated storage, transfusion

## Abstract

This study measured the changes of hemostatic activity in liquid plasma (LP) over 7 days of storage. Five canine plasma units, divided into two aliquots were evaluated: one stored refrigerated at 2–6°C as never-frozen LP and one frozen at −18°C as fresh frozen plasma (FFP). Clotting times, coagulation activities of factor (F) V, VIII, X, XI, antithrombin (AT), and von Willebrand (vWF), fibrinogen and D-dimers (DD) content were assessed before storage (baseline value), and after 12, 24, 48 h and 7 days (D7) in LP stored refrigerated, and on day 7 in FFP. At baseline median values of all factor activity were greater than 80%, and for clotting times, AT, fibrinogen and DD content, were within the canine reference range. Some hemostatic parameters changed significantly over 7 days and at the end of storage in LP. However, median activities of FV, FVIII, FX and FXI, coagulation time, AT, fibrinogen and DD content remained within reference ranges at all time points. The only exception was for vWF which median activity was lower than reference range for all storage time points. Activity of FVIII was significant lower in LP at D7 when compared to activity in FFP, with values of 62 vs. 118%, respectively. DD content showed a median value higher than reference range in FFP at D7. Despite some statistically significant changes at the end of 7-day storage period, never-frozen LP maintained median factor activities >80% for most factors. The clinical impact of the drop over time of vWF activity is unknown.

## Introduction

Canine whole blood (WB) units collected for transfusion purposes are typically centrifuged to obtain fractionated blood products, usually consisting of a unit of packed red blood cells (pRBCs) and a unit of plasma. The plasma contains therapeutic levels of functional coagulation factors and can be immediately transfused as fresh plasma or stored frozen (1–7). Plasma transfusion is an essential component of treatment for many congenital and acquired coagulopathies in dogs, such as disseminated intravascular coagulopathy, hemophilia A (deficiency of factor VIII), hemophilia B (deficiency of factor IX), von Willebrand's disease, hepatic disease, or anticoagulant rodenticide toxicity (8–10). Plasma in veterinary patients has also been used in the treatment of hypotension and to provide oncotic support in hypoalbuminemic patients during hospitalization (8, 9).

Several fresh plasma components are available for transfusion in both human and veterinary medicine. All contain coagulation proteins, but in different relative amounts (7, 11). Fresh frozen plasma (FFP) is plasma that has been separated from red blood cells (RBCs) and frozen within 6–8 h of collection (11); it can be stored frozen for 1 year at −18°C or colder and is then thawed prior to administration (11). It contains therapeutic levels of most clotting factors, adhesion proteins, fibrinogen, antithrombin, albumin, and immunoglobulins (1, 2, 4, 7) and it is the most used plasma component in veterinary medicine (12). A limiting factor for use of frozen plasma in emergency situations is the time required to thaw it. In fact, depending on the size of the bag of plasma, it can take more than 30 min to thaw plasma in a 37°C water bath (5). In addition, the thawing process has the potential to create pinholes or cracks in the plastic bag, risking contamination of plasma by dirty thawing equipment and contact with water, increasing the risk of microbial contamination of plasma units. Temperature fluctuations or excesses during thawing could also compromise coagulation factors. Frozen plasma units are fragile and rupture may occur during storage or shipment (13). Finally, as underlined in a recent study (14), the use of FFP is limited in the treatment of small dogs which may not require an entire unit of plasma.

In human transfusion medicine plasma can be separated from WB at any time during storage and stored at 1 to 6°C, without undergoing a freeze-thaw process, for up to 5 days after the expiration date of WB unit for a maximum 26 days of storage. This is known as never-frozen, liquid plasma (LP) (11) and it retains at least 50% of factor activity and thrombin generating capacity for up to 15 days of refrigerated storage (15), and has a superior hemostatic capacity for up to 26 days compared with FFP (16, 17) or was comparable to FFP in terms of hemostatic variables up to 7 days of storage (18). Never-frozen LP may overcome the limitations of FFP and allow plasma to be administered to patients with minimal delay in time-critical clinical or emergency situations. Use of never-frozen LP is associated with improved clinical outcomes (19) and has been shown to be as safe and effective as transfusion of FFP in human initial resuscitating of trauma patients (20). However, an early study in people showed a marked decrease of von Willebrand factor (vWF) in LP stored refrigerated with a final median activity of 58% after 7 days of storage (21).

Different storage temperatures might affect the coagulation factor activity in plasma units. While in veterinary medicine there have been many investigations regarding the stability of hemostatic parameters in frozen plasma (1, 3, 6, 7, 22, 23), there are few studies of the coagulation profile of never-frozen LP (2, 4, 5, 14). All veterinary studies on LP showed that storage at refrigeration temperatures had no significant effect on overall hemostatic activity of plasma. However, it is not known how storage of canine LP affects the concentration of vWF. In addition, there is no data in canine LP on activation markers for the fibrinolytic system such as D-dimers (DD), which can be a useful indicator for the quality and safety of plasma units, as the maximum storage time for plasma is also guided by the appearance of activation markers (15, 24).

The objective of this study was to measure the changes in canine hemostatic parameters over 7 days in never-frozen LP units stored at 2–6°C and to compare the value with the same plasma which had been stored frozen for 7 days as FFP. The hypothesis for this study was that LP would retain reduced, but sufficient, hemostatic capacity and unchanged DD content at the end of 7 days of storage, but that it might be deficient in vWF activity.

## Materials and Methods

### Blood Donors, Sample Collection, and Processing

In this prospective study, donor dogs were recruited from the pre-established blood donation program of the University of Milan Veterinary Transfusion Research Laboratory (REVLab) including client owned dogs. Blood units were collected with the consent of the owner of each donor dog. The protocol of this study was approved by Animal Welfare Bioethical Committee of the University of Milan (OPBA_26_2018).

WB units were collected from five privately owned, DEA 1 positive, male volunteer blood donor Golden Retrievers. All donors met the criteria for donor suitability required by Italian Ministry of Health guidelines on veterinary transfusion medicine (25). They were between 3 and 5 years of age, weighed >25 kg, were healthy and free from systemic and infectious diseases, and were not taking any medications known to interfere with coagulation testing. Blood was collected using specific canine bag collection systems (AB bags, TEC 709 Futurlab Srl, Limena, PD, Italy). The bags had been used and evaluated in a previous study (23) with CPDA-1 (citrate, phosphate, dextrose, and adenine) as anticoagulant in a ratio of 1:7 with blood.

After WB unit centrifugation in a commercial refrigerated centrifuge designed for blood separation (Rotixa 50RS – Hettich Italia Srl, Milan, Italy) within 6 h of blood collection, plasma was expressed into an attached empty satellite storage bag using a manual plasma extractor. Each plasma unit was then aliquoted into two equal units, by separation of one half, via the Luer Lock connector, into an additional empty plasma bag. One plasma unit was stored as FFP and frozen at −18°C in a dedicated plasma unit freezer (Fiocchetti Snc, Luzzara, RE, Italy), the other one unit was stored as never-frozen LP at 2–6°C in a dedicated alarmed blood unit refrigerator (Fiocchetti Snc, Luzzara, RE, Italy).

**Figure 1:**
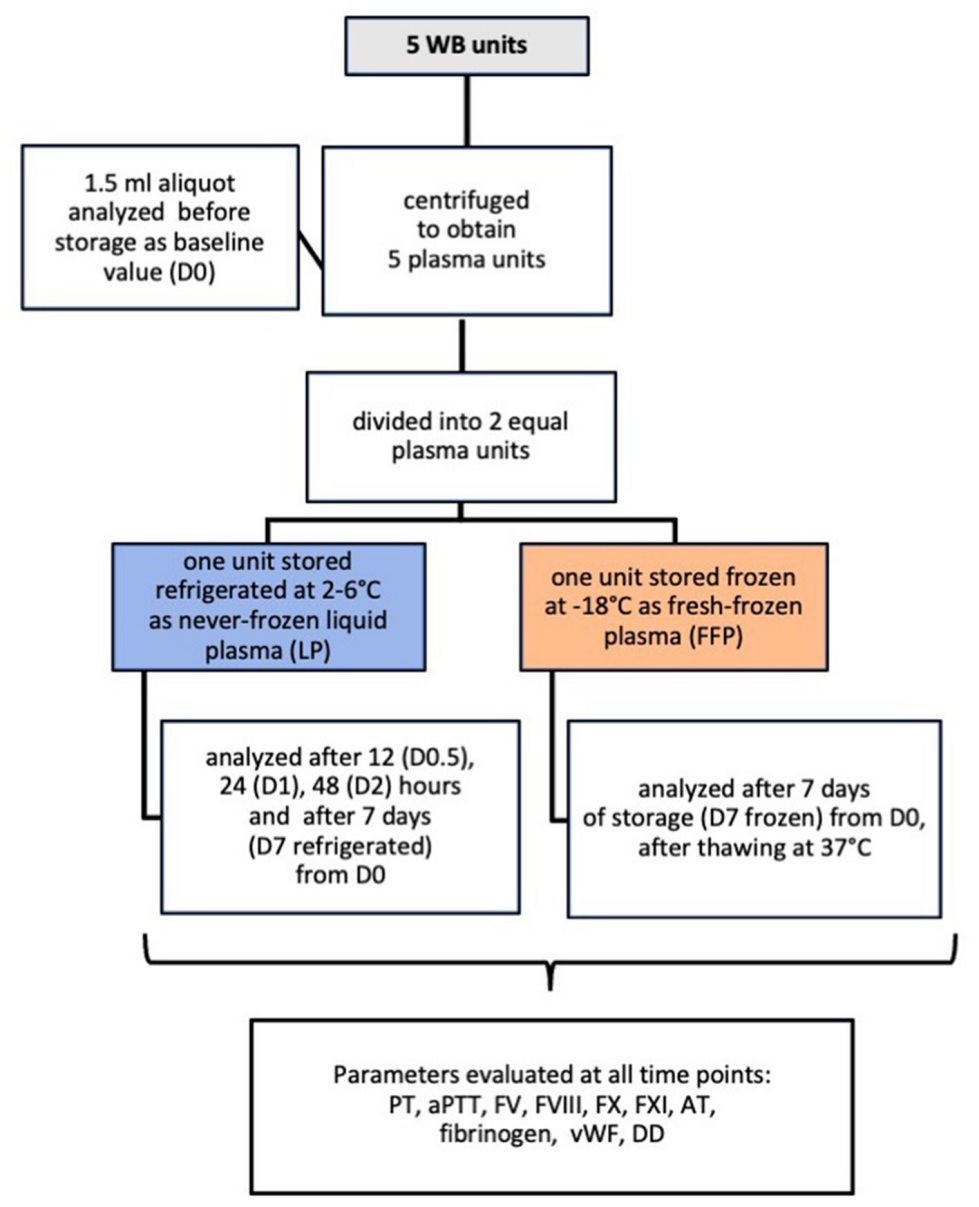
Flowchart summary of the study design in which 5 canine never-frozen liquid plasma units were evaluated for changes in hemostatic activity when stored refrigerated for 7 days compared to frozen storage.

Plasma aliquot of 1.5 ml was collected using a Luer Lock needle-free self-cleaning valve, that allowed blood sample collection whilst ensuring sterility. An aliquot was analyzed on the day of blood collection, after centrifugation and before storage (D0, baseline value), after 12 (D0.5), 24 (D1), 48 h (D2), and 7 days (D7 refrigerated) from baseline in the refrigerated never-frozen LP. After 7 days of frozen storage FFP units were thawed at 37°C, using a warm-water bath, and a 1.5 ml aliquot was sampled and analyzed (D7 frozen). Figure 1 shows a schematic flowchart of the study design.

### Laboratory Analysis

The hemostatic parameters evaluated in each plasma aliquot sample included: prothrombin time (PT), activated partial thromboplastin time (aPTT), coagulation factor activities for factor V (FV), VIII (FVIII), X (FX), XI (FXI), factor antithrombin (AT), fibrinogen, vWF and D-dimers (DD). Hemostatic proteins and parameters were measured using the STA Compact Max^®^ analyzer and related commercially available reagents (Stago Italia SRL, Milan, Italy). Canine reference ranges for hemostatic parameters were provided by the Stago manufacturer except for FV, FVIII, FX, and FXI which were taken from previous published paper (7).

### Statistical Analysis

Data were determined to be non-normally distributed using D'Agostino–Pearson test and were reported as median (95% confidence interval) and range. Analysis of variance for repeated measures (ANOVA) with Bonferroni correction for multiple comparison was performed to assess the change of hemostatic parameters over storage time, with a statistical significance set at *P* < 0.05.

The changes in median coagulation activities and times during storage were calculated for each variable, and the results at each time point were expressed as a percentage of the initial value using the following formula: % change from initial value = [(value at other times/initial value)/value at other times] x 100.

Statistical analyzes were conducted using commercial software (MedCalc^®^ Statistical Software version 20.023, MedCalc Software Ltd., Ostend, Belgium; https://www.medcalc.org; 2021).

## Results

At baseline all five canine plasma units had median values of all factor activities within the canine reference range and greater than 80%, with median baseline values ranging from 86 (FV) to 109% (FVIII). In addition, at D0 factor activities, clotting times, AT and fibrinogen content were within the canine reference range, with the exceptions of vWF and DD for which values were within the normal reference range for only 60% of plasma units (Table 1).

**Table 1:** Descriptive statistics for hemostatic activity in five paired canine plasma units stored as never-frozen liquid plasma and fresh-frozen plasma evaluated at collection (baseline value, D0), after 12 (D0.5), 24 (D1), and 48 h (D2) of refrigerated storage, and after 7 days stored refrigerated (D7 refrigerated) or frozen (D7 frozen).

**Parameter (reference range)**	**Time**	**Median (Min-Max)**	**95% confidence interval**	**% change respective to D0**
PT	D0	7.4 (7.3–7.7)	7.2–7.6	–
(7–9.8 s)	D0.5	7.6 (7.5–8.0) ^*^	7.3–7.8	+3
	D1	7.4 (7.3–7.9)	7.2–7.7	+1
	D2	7.4 (7.3–7.8)	7.2–7.7	+1
	D7 (refrigerated)	7.9 (7.6–8.2)	7.5–8.1	+6
	D7 (frozen)	7.6 (7.1–7.7)	7.1–7.8	+1
aPPT	D0	14.7 (14.1–15.7)	13.8–15.6	–
(11–16.9 s)	D0.5	14.9 (14.4–16.4)	14.1–16.0	+3
	D1	15.2 (15.0–16.8)	14.6–16.4	+5
	D2	15.7 (15.0–17.4)	14.8–17.0	+8
	D7 (refrigerated)	16.6 (15.6–18.6) ^*^	15.5–18.2	+14
	D7 (frozen)	15.2 (14.2–16.2) °	14.2–16.0	+3
FV	D0	86.0 (80.0–99.0)	79.3–98.6	–
(50–150%)	D0.5	95.0 (82.0–106.0)	81.5–105.6	+5
	D1	106.0 (83.0–110.0)	88.4–116.3	+15
	D2	99.0 (77.0–103.0)	78.4–107.1	+4
	D7 (refrigerated)	105.0 (79.0–105.0)	80.8–111.5	+8
	D7 (frozen)	105.0 (77.0–107.0)	77.2–113.5	+7
FVIII	D0	109.0 (76.0–131.0)	79.4–132.1	
(50–150%)	D0.5	87.0 (60.0–100.0) ^*^	62.9–104.2	−21
	D1	81.0 (68.0–96.0)	69.1–95.6	−21
	D2	83.0 (47.0–101.0)	55.5–106.4	−24
	D7 (refrigerated)	62.0 (33.0–69.0) ^*^	40.3–76.4	−45
	D7 (frozen)	118.0 (69.0–130.0) °	74.8–139.1	+0
FX	D0	103.0 (82.0–109.0)	81.9–112.0	
(80–175%)	D0.5	86.0 (81.0–96.0)	79.9–97.2	−8
	D1	92.0 (86.0–102.0)	84.2–102.9	−3
	D2	90.0 (78.0–99.0)	77.8–100.1	−8
	D7 (refrigerated)	84.0 (70.0–96.0) ^*^	71.3–96.6	−13
	D7 (frozen)	89.0 (78.0–97.0)	78.1–97.4	−9
FXI	D0	104.0 (88.0–108.0)	91.5–110.8	
(50–150%)	D0.5	68.0 (58.0–94.0)	56.2–91.8	−27
	D1	103.0 (85.0–110.0)	86.5–113.8	−1
	D2	97.0 (84.0–112.0)	84.3–110.8	−4
	D7 (refrigerated)	116.0 (99.0–120.0) ^*^	102.7–123.2	+12
	D7 (frozen)	119.0 (109.0–138.0)	107.6-136.3	+21
AT	D0	120.0 (114.0–130.0)	113.6–129.5	
(95–140%)	D0.5	119.0 (108.0–131.0)	108.9–130.6	−2
	D1	120.0 (112.0–130.0)	112.0–128.7	−1
	D2	112.0 (100.0–129.0)	100.9–127.8	−6
	D7 (refrigerated)	121.0 (111.0–124.0)	112.6–125.3	−2
	D7 (frozen)	120.0 (108.0–130.0)	108.1–130.2	−2
Fibrinogen	D0	174.6 (141.8–198.6)	145.5–199.2	
(100–400 mg/ml)	D0.5	169.2 (131.0–190.2)	135.8–190.2	−6
	D1	183.1 (142.9–212.4)	145.1–211.9	+3
	D2	172.2 (136.6–196.9)	135.0–200.7	−3
	D7 (refrigerated)	142.0 (122.0–172.7)^*^	122.0–167.5	−16
	D7 (frozen)	255.3 (150.7–284.2)	167.3–307.3	+37
vWF	D0	91.0 (50.0–105.0)	51.0–108.9	
(70–180%)	D0.5	69.0 (42.0–101.0)	41.2–98.7	−13
	D1	60.0 (33.0–97.0)	34.8–94.3	−20
	D2	54.0 (30.0–88.0)	32.5–87.0	−26
	D7 (refrigerated)	45.0 (26.0–62.0)	27.4–64.9	−42
	D7 (frozen)	68.0 (46.0–102.0)	44.5–96.2	−11
D-dimers	D0	0.23 (0.16–0.45)	0.10–0.46	–
(0.01–0.35 μg/ml)	D0.5	0.34 (0.12–0.39)	0.12–0.43	+1
	D1	0.23 (0.03–0.44)	0.04–0.44	−18
	D2	0.30 (0.14–0.37)	0.15–0.38	+0
	D7 (refrigerated)	0.29 (0.16–0.39)	0.18–0.41	+15
	D7 (frozen)	0.41 (0.14–0.42)	0.15–0.48	+18

Min, minimum, max, maximum; PT, prothrombin time; aPTT, activated partial thromboplastin time; FV, factor V; FVIII, factor VIII; FX, factor X; FXI, factor XI; AT, antithrombin; vWF, von Willebrand factor. Asterisks ^*^ denote a significant difference for comparison with respect to D0, circles ° denote a significant difference for comparison D7 frozen with respect to D7 refrigerated.

During storage of LP units at refrigeration temperatures most median hemostatic values did not change significantly over 7 days when compared to the value at D0, the exception being a significant increase in PT after 12 h of refrigerated storage (*P* = 0.0058). After 7 days of LP refrigerated storage (D7 refrigerated) aPTT, FVIII, FX, FXI and fibrinogen changed significantly with respect to D0. In particular, aPTT and FXI activity were statistically significantly increased (*P* = 0.0408 and *P* = 0.0001, respectively), while FVIII, FX activities and fibrinogen concentrations were significantly decreased (*P* = 0.0169, *P* = 0.0375, and *P* = 0.0228, respectively). After 7 days of frozen storage (D7 frozen), no parameter changed significantly in FFP with respect to D0. When comparing concentration and activity of hemostatic parameters between refrigerated and frozen after 7 days of storage, only aPTT and FVIII activity showed a statistically significant lower and higher value with respect to D7 refrigerated (*P* = 0.0193 and *P* = 0.0431, respectively).

**Figure 2:**
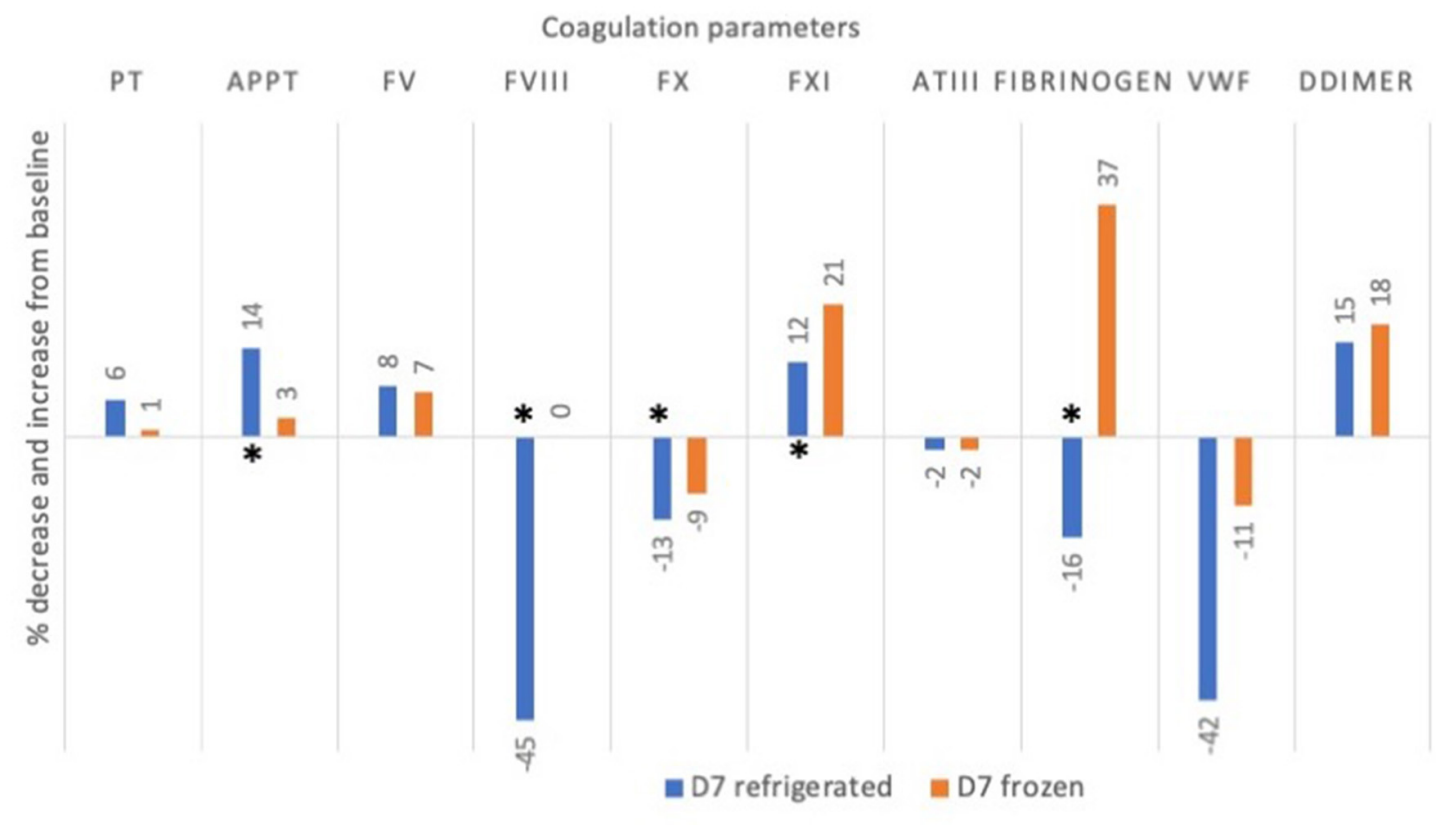
Mean percentage change (decrease or increase) in coagulation factor activities and times in 5 canine plasma units after 7 days of storage refrigerated or frozen. Asterisks ^*^ denote significant differences (*p* < 0.05) as compared to baseline value at D0. PT, prothrombin time; aPTT, activated partial thromboplastin time; FV, factor V, FVIII, factor VIII; FX: factor X; FXI, factor XI; AT, antithrombin; vWF, von Willebrand factor.

Graphical representation showing changes at D7 refrigerated and D7 frozen with respect to D0 is shown in Figure 2.

The median levels of all coagulation factors, clotting times, AT and fibrinogen at each time point remained within the reference intervals in LP stored refrigerated, with the exception for vWF for which the median value was within the reference intervals only at baseline and for DD after 7 days of frozen storage for which the median value was higher than reference ranges. At least 50% activity of all measured factors was noted on D7 refrigerated in LP, with the exception of vWF for which median activity at D7 was 45%, therefore below the lower limit of the reference range for dogs (i.e., 70%). The same decreasing trend for vWF activity was seen in FFP at D7 frozen, in which median activity was 68%.

## Discussion

The hemostatic activity of canine never-frozen LP units stored at refrigeration temperatures for 7 days was examined in our study and was compared to frozen storage in paired FFP units. A statistically significant change was found in some hemostatic parameters during and at the end of storage at refrigeration temperatures for 7 days when compared to the baseline value, but no parameters showed a significant change with respect to baseline values in the same units stored frozen. All median values for residual hemostatic parameters evaluated were within the normal reference ranges at the end of LP refrigerated storage and >50%, supporting the presumptive lack of clinical relevance of these changes. In addition, we studied for the first time the stability of vWF activity and content of DD in LP units. At the end of 7 days of LP refrigerated storage, we found a non-significant reduction of vWF median activity values which however were lower than canine reference range and <50% and a non-significant increase in DD content.

In people it has been suggested that for single factor deficiencies, such as deficiencies of FVIII or FIX, 30% activity is often needed to support clinical or surgical hemostasis (26, 27). With multiple factor deficiencies, such as after trauma, factor levels closer to 40% may be needed for hemostasis (11). There are no guidelines for the use of blood products in the veterinary field. Previous veterinary studies have used a factor activity of 50% as an acceptable threshold to support hemostasis in veterinary patients (14, 28). Another approach when considering if changes are clinically relevant is to consider whether factor levels fall inside or outside normal reference range (4, 5). As in previously veterinary studies, we consider that, as well as quantifying losses, it is also important to consider the residual coagulation factor activity in the plasma that patients receive. Thus, we considered coagulation parameters below the normal canine reference ranges and <50% to be clinically significant in plasma stored refrigerated, whereas if the coagulation parameter fell during storage but the median value remained within the reference ranges and >50%, we deemed this not to be clinically significant for the plasma recipient. Therefore, in this study we did not interpret statistically significant results as being clinically important, as statistical significance indicates the reliability of the study results, while clinical significance reflects its impact on clinical practice (29).

The data from our study reveals that in most plasma units, median levels of all coagulation factors in plasma separated on D0 were above the lower limit of the reference range, suggesting good retention of activity as previously demonstrated (23) using the same canine blood bag system as used in our study. This underlines that these canine-specifically design blood collection bags are suitable, not only for blood collection, but also to process canine blood into components as plasma units, as is usually done using human blood collection bags in canine donors (1–7).

### PT and aPTT

As in previous studies on canine plasma stored as LP at refrigeration temperatures for between 7 and 35 days (2, 5, 14), and in human LP stored for 14 and 26 days (15, 16), we also found an increase in PT and aPTT in comparison to baseline value and with respect to frozen storage, but the differences were unlikely to be clinically significant, and the values of these parameters were maintained within their reference intervals. The trend of increasing coagulation time during refrigerated storage, is not surprising, as PT and aPTT reflect the activities of multiple clotting factors, and it has been shown that a significant decrease in any factor must occur before the PT or aPTT becomes significantly prolonged (30). Therefore, the decrease in FX and fibrinogen found in our LP units could have presumably increased the PT during refrigerated storage, as PT identifies deficiencies in the extrinsic pathways of coagulation (31). The aPTT is a screening test for the intrinsic system and common pathway (31), and the marked reduction of FVIII and vWF during refrigerated storage, and to less extent, FX reduction, could have increased aPTT in LP units evaluated in our study.

### FV

Our study found a non-significant increase in FV activity during and at the end of storage in both LP and FFP. Certain coagulation factors and hemostatic proteins such as FV are heat labile. Unstable proteins decrease within plasma over time under certain storage conditions, showing significant decreases (to 39% of their mean initial activities) during storage of LP for 26 days (16). However, other studies demonstrated that FV did not change significantly during 5 days of storage (15), and remained stable in LP for up to 14 days of storage (21).

In our and previous veterinary studies, no significant differences for FV were noted between LP and FFP after 7 days of storage (5) and up to 35 days of storage with a median final value that remained >50% (14). In our study FV remained stable for up 7 days in LP and was similar in FFP. Therefore, storage at refrigeration temperatures maintains therapeutic levels of FV. In addition, FV appears to be stable during refrigerated storage for 7 days as compared to frozen plasma at D7. This was not surprising as Urban et al. found no reduction of FV in frozen plasma after storage for 5 years (4). Results of our and previous veterinary studies on stored canine plasma underline that, in contrast to some human literature (16), FV seems to be a stable plasma coagulation factor in canine LP units.

### FVIII

Our study found a significant decrease of FVIII during and at the end of LP storage, while activity in FFP at D7 was increased with respect to baseline values, and showed a significant increase respect to 7 days of refrigerated storage. Human and veterinary literature states that FVIII suffers the greatest degree of degradation in stored plasma as it is one of most heat labile, unstable proteins which decreases within plasma over time under refrigerated and frozen storage conditions (1, 32, 33). In human LP the activity of FVIII exhibited small (11%), but significant, decreases over the first 5 days of refrigerated storage with a final mean activity of 50% after 30 days of storage (15). When LP was stored for 26 days, FVIII significantly decreased, to 60% of its mean initial activity, during storage (16). FVIII fell slowly to about 50% of its original level between the 7th and 14th day of refrigerated storage of human LP (21). In agreement with previous studies, FVIII decreased by 45% with respect to baseline values in our stored LP units. These results confirm that FVIII was one of the most heat labile factors, as reported in a study on frozen plasma (1, 4) and in examination of citrated plasma collected for diagnostic purposes in which FVIII was unstable after only 2 days from collection when stored refrigerated, even if the factor activity remained in the normal reference intervals when stored for up 4 days (34). In contrast to our finding that FVIII showed a significant reduction (of −45%) in LP units, a previous veterinary study showed that FVIII activity decreased only approximately 15% in the first week of LP refrigerated storage (5). Possible reasons for the difference in coagulation factor activities are that the refrigerated LP unit samples in that previous study (5) were shipped frozen to a reference laboratory and then stored frozen for later batch analysis. Therefore, before analysis, LP was frozen and thawed. Human studies have demonstrated that after freezing, LP behaves like thawed FFP (16).

It has been postulated that instability of some coagulation factors at low temperatures may be the result, at least in part, of cold-induced activation of proteolytic enzymes from white cells (WBC) and platelets (PLT). These enzymes may be partially responsible for the slow degradation of many clotting factors (including factors VIII, IX, and XI) when plasma is stored at 4°C (21, 35). The canine plasma units evaluated in this study were obtained from non-leukoreduced WB units, therefore they could contain all the whole blood WBC and PLT.

Unlike FV, FVIII appears therefore to be an unstable coagulation plasma factor. Although FV and FVIII are homologous coagulation cofactors, their B domain differs in man, as FV has 25 potential N-linked glycosylation sites, but FVIII has 18 (36). Some authors (37) hypothesize that this difference may explain why FV appears to be more heat stable than FVIII. However, even though the B domain constitutes ~40% of the FVIII molecule, and different regions of B domain might participate in expression, intracellular transport, secretion and stability to various degrees, the functional characteristics of its specific regions are still obscure and its function has not been well-characterized (38). Additional studies are needed to understand whether changes in the B domain make the factors more or less heat stable and whether this could apply also to plasma in dogs.

Despite the significant loss of FVIII at the end of 7 days of refrigerated storage of our LP units, median values for this factor remained within the normal range in all but one unit, suggesting that these findings are not likely to be clinically relevant. This finding was previously reported by Urban et al. who found that although FVIII activity was significantly lower in refrozen plasma at −30°C after 5 years of storage than in fresh plasma, the frozen plasma remained hemostatically active as evaluated by thromboelastography (4).

### FX

Although in our study FX decreased significantly at D7 refrigerated compared at D0 in LP, the median value remained within the reference range at the end of storage, underlining that this finding is not likely to be clinically relevant. This trend was in agreement with findings in a previous study on canine LP, in which FX concentration decreased significantly from day 0 to day 7, but with the median concentrations remaining in the normal range (5). FX activity level was also found to be stable after 7 days of refrigerated storage in a recent study on canine LP and showed a significant activity reduction only at 35 days of storage, but with activity that remained >50% (14). In an early human study, no decrease in FX was observed in LP up to 14 days of storage (21) and it was found to be stable over 26 days storage (16). Another study found that FX concentration was one of the least variable factors over the 30-day evaluation, and the minimal changes seen during storage were not significant (15).

### FXI

We found a statically significant increase in FXI in LP at the end of storage when compared to baseline value. However, the median values for this factor were within normal reference range suggesting a lack of clinical significance of these changes, despite significant intra- and intergroup differences at some time points. FXI was studied in a recent veterinary study, in which, its activity increased significantly on the third day of storage, but then decreased progressively to the 21st day before increasing at day 28 and decreasing by day 35. However, its activity was <50% only in FFP on day 7 of storage (14). Similarly to the findings in our study, FXI in that study showed significant intra- and intergroup differences at some time points, the reason and significance of which are unknown. In human LP there was no significant change in FXI activities during the first 5 days of storage (15) and FXI was stable up to 26 days of storage (16).

### AT

Our study found a slight non-significant decrease of AT activity during and at the end of storage in both LP and FFP units. Only one recent veterinary study has investigated the effect of refrigerated storage on AT concentrations in canine LP in which level of this protease inhibitor remained >50% for up to 35 days of refrigerated storage and was higher than the median level found in paired FFP stored for the same time (14). AT was previously studied in canine frozen plasma, where frozen storage at −70°C for 6 months was found to cause a decline in AT activity (39). However, a more recent study suggested that AT activity in plasma collected for transfusion and stored frozen for 5 years was biologically equivalent to plasma at the time of collection (4).

With respect to AT in human LP, early and recent studies showed that there was no significant degradation of functional AT for up to 30 days of refrigerated storage (15, 21). Coagulation inhibitor AT remained stable during 26 days of storage compared with values at baseline, with a final value increase of 3% with respect to D0 (16). In agreement with previous veterinary and human studies, AT content in both LP and FFP units in our study showed a minimal, non-statistically nor clinically significant reduction of 2% when compared to the baseline value at D0, and along with recent findings (14) shows that refrigerated never-frozen LP could be a source of AT in the dog.

### Fibrinogen

Our study found a statistically significant decrease in fibrinogen content in LP units at final refrigerated storage time, while the fibrinogen content was increased in FFP units at D7. Previous veterinary studies showed that refrigerated storage for 14 and 30 days of canine LP obtained for transfusion purposes had no significant effect on fibrinogen content when compared to the baseline value at D0 (5) or to storage at −30°C (2). In another veterinary study, fibrinogen was significantly lower in plasma frozen for 5 years than in fresh plasma. However, these findings are not likely to be clinically relevant because the values were still within the reference interval and the plasma appeared to be hemostatically active when evaluated by thromboelastography (4). Fibrinogen concentrations were significantly less by day 14 of storage for canine LP, compared with the baseline in another more recent study, but decreases were small, and concentrations did not significantly differ between LP and FFP (14). Fibrinogen was stable in most human studies on LP, showing no significant change during the first 5 (15), 14 (21), 26 (16) and up to 28 days of storage (17). As previously demonstrated in these veterinary and human studies, we also showed a non-significant change in fibrinogen content in our LP units, with levels at the end of storage within their reference ranges even if there were statistically significant differences when compared to the baseline value. It should be pointed out that fibrinogen content increased by 37% in our FFP units at the end of 7 days frozen storage. No explanation can be offered for this, as fibrinogen maintains its content and function when stored at −20°C for up 6 months, showing only a minimal increase from baseline value (40) and it is possible that an analytical error could be the explanation. However, because median fibrinogen at final storage was within normal reference range, these changes were unlikely to be clinically important.

### vWF

Our study demonstrates, for the first time, how vWF activity changes during refrigerated storage of never-frozen LP collected for transfusion. vWF activity showed a gradual and non-statistically significant reduction over 7 days of refrigerated storage when compared to the baseline value, with median final values lower than canine reference ranges. The same result, although less pronounced, was seen in FFP units stored for the same length of time. The drop of median vWF activity to below the reference range might be due not only to the different storage temperature, but also because vWF values at D0 for some plasma units (40%, 2/5 units) were already below the lower limit of the normal canine reference range for vWF. In addition, vWF activity varied between our five produced plasma units and in only 60% of these units were baseline values within the reference range. As previously reported in human (17) and veterinary studies (1, 6, 14, 23), our results indicate wide inter-donor variability in single-donor plasma units with regard to the composition of individual factors in a unit of plasma, with particular reference to vWF activity. We tried to re-analyze the results excluding the two units with lowest vWF activity at D0 (data not shown), but the final results did not change.

Suitable blood donors for inclusion in our blood donor program are selected following the guidelines on veterinary transfusion medicine of the Italian Health Minister (25). This requires, among other characteristics, that a suitable blood donor should be annually tested and have a normal coagulation profile consisting of PT, aPTT and fibrinogen level evaluation. However, as shown in this study, this didn't guarantee that all coagulation parameters were within the normal reference ranges, as 2/5 donors produced a plasma unit with vWF below the lowest reference range. This bring us to suggest that blood donors be screened for selected factors when plasma is collected for the specific use of replenishing selected coagulation factors in a deficiency such as vWF. In addition, attention should also be paid to the blood collection technique as vWF activity could be affected by techniques that prevent platelet activation and clot formation, conditions that deplete and degrade vWF in the sample specimen (41). However, we did not evaluate this aspect during blood collection in our study.

In both LP and FFP, after 7 days storage time, median vWF activities were below the lower limit of canine normal references range, and with a median activity <50% in LP (45 in LP vs. 68% in FFP). Therefore, based on these results, frozen storage seemed to preserve better the activity of this coagulation factor. In human LP the measured activity of vWF has shown contrasting results. An early study showed a marked decrease of vWF in plasma stored refrigerated with a final median activity of 58% after 7 days of storage (21). A more recent study demonstrated the most variation over the 30-day storage period, although vWF did not change significantly after 5 days of storage (15). Another study noticed an increase in vWF in some plasma units after 26 days storage of LP, most likely caused by cold-promoted clotting activation and/or release of vWF from activated platelets (16).

### DD

Our study found a non-significant increase of DD in both LP and FFP units at final storage time, with median value in FFP at D7 that was higher than canine reference ranges. DD is one test for hemostatic activation and fibrinolysis (31) as they form when thrombin initiates the transformation of fibrinogen to fibrin. Therefore, DD are markers of coagulation and complement activation. Activation processes have significant impact on the quality and safety of plasma units, as the maximum storage time for plasma is also guided by the appearance of activation markers (15, 24). DD have already been used in previous human and veterinary studies, for example to evaluate the effect of leukoreduction on plasma quality (42, 43). However, to the authors' knowledge, this study evaluated DD for the first time in canine never-frozen LP. In our study DD concentrations showed a non-significant increase after 7 days of storage in LP, similarly to that reported in human studies on LP where DD remained stable during 26 or 42 days of refrigerated storage (16, 24) or showed progressive and slight significant reductions, but without a meaningful change in their levels observed over 30 days of refrigerated storage (15). These findings underline the high quality of canine never-frozen LP at the end of 7 days storage. In our study DD also showed a non-significant increase in FFP, however the median value in FFP after 7 days of frozen storage was higher than the canine normal range. The observation of a greater increase of DD in FFP than in LP at D7 could be consistent with a greater cold-induced activation and subsequent fibrin degradation during storage, which happened at a low rate during refrigerated storage.

### Limitations

The main limitation of this study was the small sample size of evaluated plasma units, that may not have been representative and this precludes generalization of the results to the entire canine population. However, the plasma from the same donor was prepared and stored as FFP or LP units, for direct comparisons. In addition, only plasma samples from male Golden Retrievers were evaluated, and some human studies have demonstrated a relationship between ABO blood group, gender, race and hemostasis (15, 24, 44). A more diverse breed population would be more representative of a canine community-based blood bank. Finally, we tested only for the presence of coagulation factors, but not biological activity and no *in vivo* testing was done. Therefore, *in vivo* studies are indicated to confirm our findings. Despite these limitations, results of our study confirmed most of the previous findings on hemostatic activity of canine stored LP and adds new insight into the coagulation properties of never-frozen LP that could be a precious resource in emergency situations, where time for plasma thawing is a limitation.

## Conclusion

In conclusion, this study showed that canine plasma can be stored as never-frozen LP at refrigerated temperatures for an initial period of up to 7 days with levels of FV, FVIII, FXI, AT, and fibrinogen remaining within their reference ranges and >50%. Only vWF median values activity dropped below 50% at the end of storage, clinical significance of which is unknown. LP units stored refrigerated for 7 days can be especially useful for certain emergency settings where time for plasma thawing is a limitation. Additional studies are needed to understand if the low content of vWF in LP after 7 days of storage could be still sufficient for emergency treatment of a vWF deficiency patient.

## Data Availability Statement

The original contributions presented in the study are included in the article/supplementary material, further inquiries can be directed to the corresponding author/s.

## Ethics Statement

The study was carried out with client-owned dogs after approval by the University of Milan Animal Welfare Bioethical Committee (OPBA_26_2018) of Milan and with owner informed consent. Written informed consent was obtained from the owners for the participation of their animals in this study.

## Author Contributions

ES, RP, and DP: acquisition of data, analysis and interpretation of data, contributions to conception and design, and acquisition of clinical data. LB: contributions to laboratory test. ES: writing—original draft preparation. ES, RP, LB, and DP: writing—review and editing. DP: project administration. All authors read and approved the final manuscript.

## Conflict of Interest

The authors declare that the research was conducted in the absence of any commercial or financial relationships that could be construed as a potential conflict of interest.

## Publisher's Note

All claims expressed in this article are solely those of the authors and do not necessarily represent those of their affiliated organizations, or those of the publisher, the editors and the reviewers. Any product that may be evaluated in this article, or claim that may be made by its manufacturer, is not guaranteed or endorsed by the publisher.
